# The role of the complosome in health and disease

**DOI:** 10.3389/fimmu.2023.1146167

**Published:** 2023-03-10

**Authors:** Fang Xiao, Jixu Guo, Stephen Tomlinson, Guandou Yuan, Songqing He

**Affiliations:** ^1^ Division of Hepatobiliary Surgery, The First Affiliated Hospital of Guangxi Medical University, Nanning, Guangxi, China; ^2^ Department of Microbiology and Immunology, Medical University of South Carolina, Charleston, SC, United States; ^3^ Key Laboratory of Early Prevention and Treatment for Regional High Frequency Tumor (Guangxi Medical University), Ministry of Education, Nanning, China; ^4^ Guangxi Key Laboratory of Immunology and Metabolism for Liver Diseases, Nanning, Guangxi, China

**Keywords:** complement, complosome, T cells, monocytes, tumor cells

## Abstract

The complement system is one of the immune system’s oldest defense mechanisms and is historically regarded as a liver-derived and serum-active innate immune system that ‘complements’ cell-mediated and antibody-mediated immune responses against pathogens. However, the complement system is now recognized as a central component of both innate and adaptive immunity at both the systemic and local tissue levels. More findings have uncovered novel activities of an intracellularly active complement system—the complosome—that have shifted established functional paradigms in the field. The complosome has been shown to play a critical function in regulating T cell responses, cell physiology (such as metabolism), inflammatory disease processes, and cancer, which has amply proved its immense research potential and informed us that there is still much to learn about this system. Here, we summarize current understanding and discuss the emerging roles of the complosome in health and disease.

## 1 Introduction

The complement system is composed of more than 50 plasma and cell-bound components, and comprises a major arm of the immune system. From its identification over a hundred years ago, our understanding of the complement has completely changed from what was thought to be a blood-borne antimicrobial system to a global and local regulator of immunity and tissue homeostasis (1, 2). A long-held belief was that complement proteins are synthesized by various cell types, principally hepatocytes, and were operative only in the extracellular milieu. However, the discovery of functionally active complement proteins, such as C3 and C5, in the intracellular compartment of T cells initiated a new chapter for complement research (3–6). This intracellular complement system termed the ‘complosome’ has since been identified in a variety of cell populations, including T cells, monocytes, macrophages, neutrophils, tumor cells, epithelial cells, and other immune and non-immune cells. There is increasing recognition that the complosome contributes significantly to normal cell and organ development, homeostasis, and tissue repair (2, 7–9). The intracellular complement system also engages in crosstalk with intracellular innate sensor systems, such as the Pyrin domains-containing protein 3 (NLRP3) inflammasome in cell metabolic pathways (5, 10, 11). Our growing understanding of the complement system, particularly the complosome, in relation to health and disease is opening up new research avenues and potential therapeutic strategies.

## 2 Conventional complement cascades

**Figure 1:**
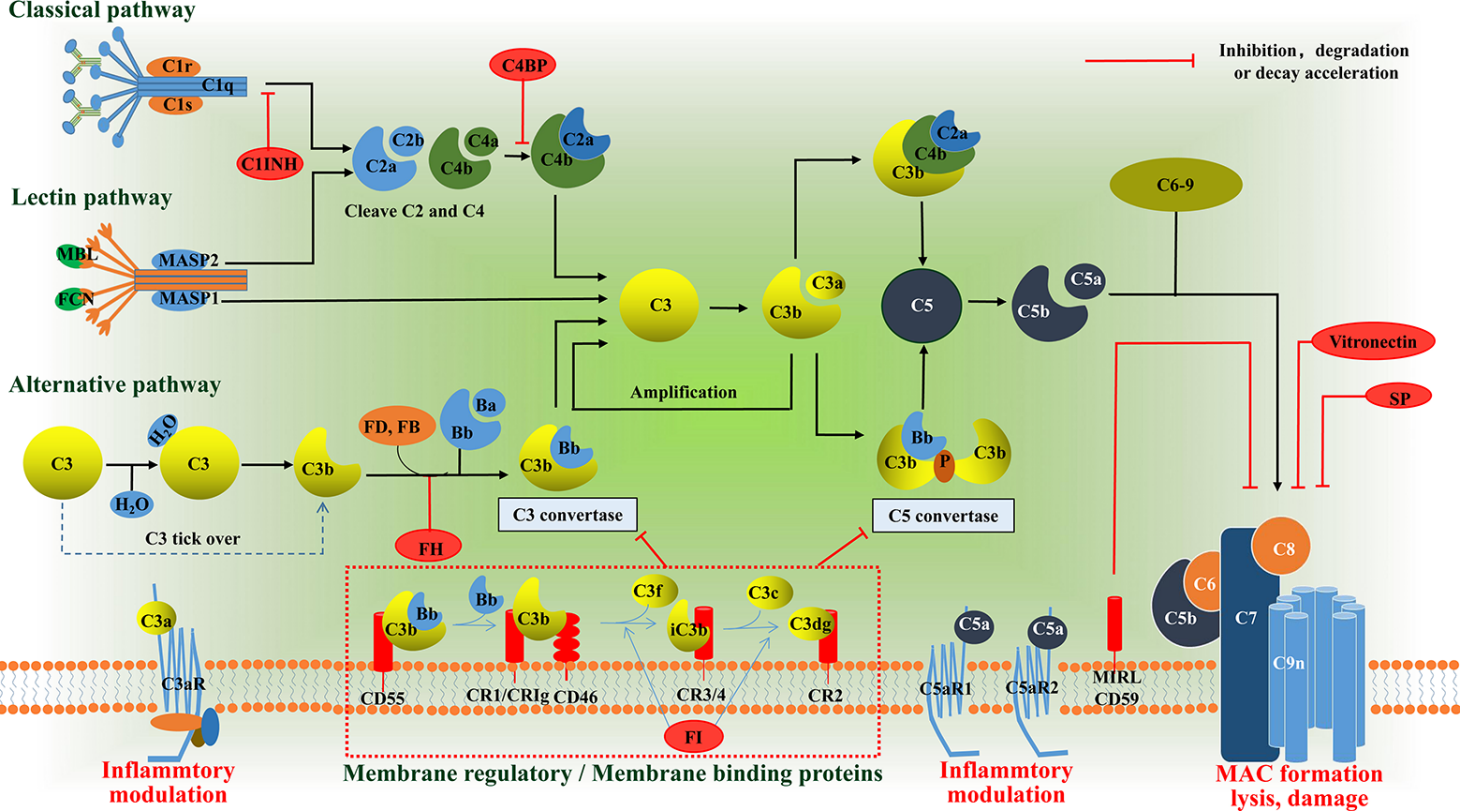
Schematic diagram of the complement system. The complement system can be activated *via* the classical pathway (CP), the lectin pathway (LP), or the alternative pathway (AP). These three pathways have their own pattern in forming C3 convertase: C4b2a or C3bBb. The C3a binds its anaphylatoxin receptor the C3aR, while C3b can induce phagocytosis of opsonized targets and its further degradation products, iC3b, C3c and C3d/C3dg are able to bind various complement receptors. C3b can also bind to the former C3 convertase, which then results in formation of the C5 convertases: C4bC2aC3b or C3bBbC3b. The C5 convertases cleaves C5 into the C5a and C5b. The C5a binds its anaphylatoxin receptor the C5aR1 and C5aR2, whereas C5b, C6, C7, C8 and up to 16 molecules of C9 bind together to form the MAC, which forms a channel on the surface of targeted cells and causes cells to swell and lysis. In addition to the rate-limiting effect of the C3 and C5 convertase, there are also soluble regulatory proteins (i.e., C1INH, C4BP, and FH) and membrane regulatory proteins (i.e., CR1, CD46, and CD55) that regulate the activation of complement cascades. The anaphylatoxins C3a and C5a mediate inflammatory mudulation through binding to their receptors, respectively. MBL, mannose binding lectin; MASP, MBL associated serine protease; FD, factor D; FB, factor B; FH, factor H; C1INH, C1 inhibitor; FI, factor I; C4BP, C4b-binding protein; CR, complement receptor; MAC, membrane attack complex, SP, S protein.

Research into the complement system has undergone something of a renaissance over the past 15 years, principally due to the recognition that it plays a key role in many disease processes and, with it, the promise of anti-complement therapies. Complement proteins, in general, exist in inactive forms in body fluid, and are activated to produce proinflammatory mediators, opsonins, and cytolytic membrane attack complexes (MAC). Activation of complement normally occurs *via* one of three pathways (**Figure 1**): The classical pathway (CP), the lectin pathway (LP), and the alternative pathway (AP) (12, 13). All three pathways converge with the cleavage and activation of C3, leading to the generation of C3 and C5 convertase enzymes that amplify the activation cascade. Activation of the CP usually occurs following the binding of the C1 complex to Fc regions of complement-fixing antibodies, leading to cleavage of C2 and C4, and resulting in the formation of the CP C3 convertase (C4b2a) (14). The LP follows a similar activation mechanism, except that it employs a mannose-binding lectin/carbohydrate recognition system with different C2 and C4 cleaving enzymes (15). In the AP, soluble C3 is spontaneously hydrolyzed through a “C3 tick over” mechanism to form C3b(H_2_O), which results in exposure of a site that recognizes factor B and leads to the generation of the AP C3 convertase, C3bBb (16). However, it is initially activated, and the next step in the cascade occurs when either the CP or AP C3 convertase cleaves C3 into C3a and C3b. The larger C3b fragment can associate with additional C3 convertases to form CP and AP C5 convertases (C4b2a3b/C3bBb3b). These C5 convertases cleave C5 into C5a and C5b. The larger C5b fragment associates with C6 to form C5b6 which associates with cell membranes, followed by the sequential recruitment of C7, C8 and multiple C9’s to form the membrane penetrating and cytolytic MAC (17). There are, by necessity, several mechanisms in place to prevent uncontrolled activation of complement. In addition to the rate-limiting effect of the C3 and C5 convertase, there are also soluble regulatory proteins (i.e., C1INH, C4BP, and FH) and membrane regulatory proteins (i.e., CR1, MCP/CD46, and DAF/CD55) that regulate the activation of complement cascades (18). The intermediate products, C3a and C5a, which are anaphylatoxins, bind to corresponding transmembrane receptors and play an inflammatory role. As for C3b, it also can induce phagocytosis of opsonized targets as well as serves to initiate the amplification loop in the AP. The conventional complement regulatory cascades are shown in **Figure 1**. Activation of intracellular complement, the complosome, and its interaction with extracellular complement systems have received extensive focus since its discovery (3). The complosome not only affects how we interpret traditional complement cascades, but it also has the capacity to reconstruct the framework of complement cascades (19). Here, we aim to summarize current understanding and discuss the emerging roles of the complosome in health and disease.

## 3 The history of the complosome

In 2013, a study on complement subverted our perception of complement activation and showed that complement is not confined to the extracellular space but can also occur intracellularly. This landmark study discovered that the intracellular reserves of C3, C3aR (located on lysosomes), and cathepsin L (CTSL) are present in human CD4+ T cells and are essential for maintaining T cell homeostasis and mediating effector differentiation. Furthermore, the intracellular C3 stores and ‘Tonic’ intracellular C3a generation have also been shown to occur in monocytes, neutrophils, CD8^+^ T cells, B cells, epithelial cells, endothelial cells, and fibroblasts, implying that intracellular complement activation might be of broad physiological significance (3). Inspired by this exciting discovery, the novel concept of ‘complosome’ was first introduced in 2014 by Claudia Kemper’s team, which was somewhat analogous to the inflammasome (19). Then other studies have shown that another important complement component, C5, also exists intracellularly in T cells and plays a role in regulating Th1 response, reactive oxygen species (ROS) generation, and NLRP3 inflammasome activation (5). Another important study demonstrated the movement of a portion of the intracellular C3 (also known as C3(H2O)) pools from the extracellular milieu (serum/blood) into T and B cells (20). The hydrolyzed form C3(H2O) was quickly taken up and mostly returned to the extracellular environment within 24 hours, and this mechanism was dubbed the recycling pathway for extracellular-derived C3 (20). In 2017, another complement component, Factor H (FH), has been found to be internalized (bound to nucleosomes) by apoptotic cells (Jurkat T cells), where it directly binds to CTSL rather than being degraded. The FH functions intracellularly as a cofactor for the CTSL-mediated cleavage of C3 (21). Emerging evidence supports that a variety of complement components, receptors, and regulators appear to be present inside T cells and other cells, just like the inflammasome, therefrom, the ‘complosome’ has ignited excitement in the field. An entire session at the XXVI International Complement Workshop in 2016 was dedicated to the complosome. Despite the robust evidence that has been accumulated to date, the veracity and relevance of the complosome are still being questioned. Even though there is controversy surrounding the complosome, scientists in the field still believe that this new area of research will remain mainstream for the near future, as it would help to understand the crosstalk between intracellular complement proteins and inflammasome molecules, as well as other intracellular proteins underlying in health and disease (22–24).

## 4 Complosome in immune cells

### 4.1 T cells

**Figure 2:**
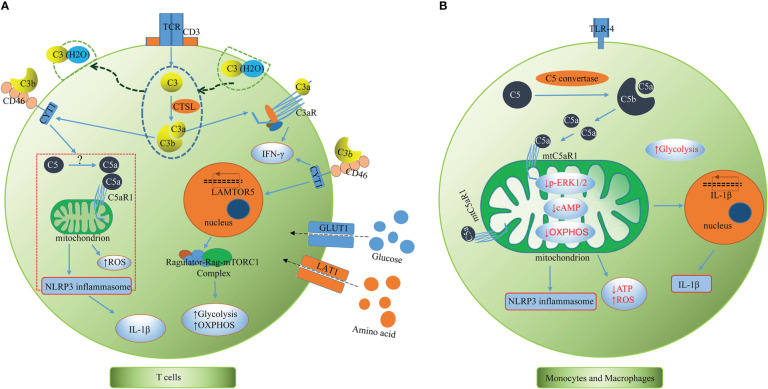
The complosome in T cells and monocytes and macrophages. **(A)** T cells. T cells have intracellular store of C3 (partly taken up from serum as C3(H2O)), C5 and CTSL. CTSL continuously cleaves intracellular C3 into bioactive C3a and C3b. Upon TCR stimulation, the intracellular C3 and activation fragments C3a, C3b and CTSL translocate to the cell surface where C3a and C3b signal in an autocrine activation though C3aR and CD46, respectively, to to induce Th1 immunity and IFN-γ production. The CD46 activation drives expression of LAMTOR5, LAT1 and GLUT1, which promotes mTORC1 activation, nutrient influx, glycolysis, and OXPHOS. CD46 also induces intracellular C5 activation and C5a generation *via* yet unknown mechanisms. The intracellular C5a interacts with the mitochondrially expressed C5aR1, resulting in mitochondrial ROS production, intrinsic NLRP3 inflammasome assembly and activation, maintenance of IFN-γ secretion, and subsequent mature IL-1β secretion during T cell migration into inflamed tissues. CTSL, cathepsin L; TCR, T cell receptor; LAMTOR5, late endosomal/lysosomal adaptor and MAPK and mTOR activator 5; GLUT1, glucose transporter; LAT1, large neutral amino acid transporter 1; mTOR, mammalian target of rapamycin; OXPHOS, oxidative phosphorylation; ROS, reactive oxygen species. **(B)** Monocytes and macrophages. Intracellular complement C5 is cleaved and generates C5a *via* intracellular C5 convertase (C3bBbC3b). C5a/C5aR1 signaling on mitochondrial membranes alters mitochondrial activity, reduces mitochondrial ERK1/2 phosphorylation, cAMP formation and shifts ATP production *via* reverse electron chain flux toward ROS production and aerobic glycolysis, promoting IL-1β gene expression and processing of bioactive Il-1β upon DAMP sensing. cAMP, Cyclic Adenosine monophosphate; ATP, adenosine triphosphate; ROS, reactive oxygen species; DAMP, damage-associated molecular pattern.

To date, Claudia Kemper’s team has taken the lead in conducting most of the research on the complosome and have mostly described it in human T cells, highlighting its connection to inflammasome activation and immunometabolism (**Figure 2A**). As mentioned above, both intracellular C3 and C5 system, together with their interaction with the inflammasome, are indeed required for effective Th1 immunity (3, 5). The complosome is required to induce metabolic reprogramming of immune cells, including increased glycolytic flux and oxidative phosphorylation (OXPHOS), which promote the secretion of the proinflammatory cytokine IFN-γ (25). In resting T cells, CTSL constantly cleaves intracellular C3 into bioactive C3a and C3b, and the intracellular C3a/C3aR engagement maintain T cell survival *via* low-level mTOR induction (3). Upon toll like receptor activation, the entire intracellular C3 system translocates rapidly to the cell surface, where C3a and C3b signal in an autocrine manner *via* their receptors, C3aR and CD46, respectively, to induce Th1 immunity and IFN-γ production. Moreover, during T cell activation, CD46-mediated signals permit nutrient influx *via* expression of the large neutral amino acid transporter 1 (LAT1) and glucose transporter 1 (GLUT1). Autocrine activation of CD46 simultaneously drives induction of late endosomal or lysosomal adaptor and MAPK and mTOR activator 5 (LAMTOR5), which facilitates the assembly of the Amino Acid-sensing Ragulator-Rag-mTORC1 complex and promotes OXPHOS and glycolysis, two processes necessary for IFN-γ production and Th1 lineage commitment (4). This demonstrates a crucial connection between the complement system and immune metabolism guiding human CD4+ T cell effector function. Similarly, upon TCR and CD46 co-stimulation, the intracellular C5a interacts with the mitochondrially expressed C5aR1, resulting in mitochondrial ROS production, intrinsic NLRP3 inflammasome assembly and activation, maintenance of IFN-γ secretion, and subsequent mature IL-1β secretion during T cell migration into inflamed tissues. Additionally, CD4+ T cells express C5aR2 on their surface and intracellularly, and both secreted C5a and the desarginated version of C5a (C5a-desArg) interact with C5aR2 in an autocrine manner. C5aR2 negatively regulates the C5aR1-driven NLRP3 inflammasome activity, which results in a reduction in IFN-γ production, a switch to IL-10 secretion, and a suppression of Th1 responses (5). Moreover, the complosome also participates in the contraction phase of T cell responses. Following effective Th1 induction, CD46-mediated signals interacts with the interleukin (IL)-2 receptor, reduces glycolysis and OXPHOS, promotes IL-10 production in these cells, which induces them to enter a self-regulative contraction phase (8). Moreover, human cytotoxic CD8+ T cells (CTLs) also harbor a complosome system. The TCR and autocrine CD46 costimulation drives IFN-γ production, nutrient influx, and cytotoxic activity in these cells. Interestingly, in CTLs, aside from a strong OXPHOS induction, CD46 is also a potent inducer of fatty acid synthesis. Although CD8+ T cells express NLRP3, the inflammasome is not required for normal IFN-γ secretion or cytotoxicity in CTLs (26, 27). The activated CD8+T cell can also take up C1q, another complement protein, and restrains the response to self-antigens by modulating the mitochondrial metabolism *via* gC1qR and a yet unknown mechanism (28). Given what we presently know about the complosome in T cells, it is possible that the complosome also plays a role in the development of T cell memory and/or tissue residency, these subjects are being explored currently (29).

This novel pathway of complosome in T cells contributes greatly to disease. For example, Juvenile idiopathic arthritis (JIA) patients’ T cells have increased intracellular C3. *In vitro* treatment with a cell permeable CTSL inhibitor can normalize the hyper-active intracellular C3 activation and decrease IFN-γ production in patients’ T cells (3). Intracellular C3 system dysregulation contributes to human autoimmune diseases such as scleroderma, systemic lupus erythematosus (SLE), RA, and multiple sclerosis, as pathological intracellular C3 hyperactivation contributes to Th1 hyperactivity in these autoimmune conditions (4, 30). Individuals lacking CD46 expression or C3 secretion by T cells have diminished Th1 immunity (but normal Th2 and TH17 responses) and suffer from recurrent infections (31, 32). Patients with leukocyte adhesion deficiency type 1 (LAD-1) have reduced C3 transcripts and diminished effector activities in immune cells, which have been shown to be rescued proportionally by intracellular C3 provision. It was also shown that C3 transcription is LFA-1 (lymphocyte function-associated antigen 1)-dependent, and perturbations in the LFA-1-C3-axis contribute to primary immunodeficiency (33).

### 4.2 Monocytes and macrophages

The complosome plays a crucial orchestrating role in cell metabolic processes that regulate T cell effector responses, but the role of the complosome in myeloid immune cells was unknown for several years. There was preliminary evidence showing human monocytes produce IL-1β during infections by relying on intrinsic C3a activity (10). Macrophage populations express C3a receptor (C3aR) intracellularly (34). M2 macrophages highly express intracellular C3/C3b, which modulates the proinflammatory profile during endoplasmic reticulum (ER) stress (35). In general, studies regarding this topic were relatively scarce until recently, when Kemper’s team published a remarkable study that revealed that human monocytes and macrophages continuously synthesized C5 and produced C5a intracellularly *via* an intrinsic intracellular C5 convertase (**Figure 2B**). C5a/C5aR1 signaling on mitochondrial membranes was shown to alter mitochondrial activity and shift adenosine triphosphate (ATP) production *via* reverse electron chain flux toward ROS production and aerobic glycolysis, which not only promoted IL-1β gene expression, but also processing of bioactive Il-1β upon damage-associated molecular pattern (DAMP) sensing in monocytes and macrophages (36). These results further elaborated previous findings that C5a augments physiologic inflammasome responses (11, 37) from the perspective of intracellular complement. Unlike the previous conclusions that cell surface anaphylatoxin receptors are indirect regulators of mitochondrial activity (38), evidence has demonstrated that cellular mitochondrial C5aR1 (mtC5aR1) activation can directly reduce mitochondrial ERK1/2 phosphorylation, as well as ATP and cAMP formation and OXPHOS, simultaneously increasing ROS production and glycolysis, thereby establishing a direct link between cellular C5a/C5aR1 signaling and cell metabolic pathways located within the mitochondria. Upon mtC5aR1 blockade, IL-1β suppression was only observed in M2 macrophages, but not M1 macrophages. Conversely, mtC5aR1 inhibition reduced IL-10 production in differentiated M1 macrophages and not M2 macrophages, suggesting that there is still much to learn regarding the interaction between the complosome and macrophages. Furthermore, intracellular factor B was proposed to play a role in this process in macrophages. In diseased states, mice benefited from conditional knockout of C5ar1 in myeloid cells in models of crystal-triggered sterile inflammation (folic acid–induced kidney injury and atherosclerotic cardiovascular disease (ACVD) (36). This research has greatly increased our current knowledge on the complosome in myeloid immune cells and it is of great significance to the follow-up research.

### 4.3 Other immune cells

There is growing evidence that the complosome exists and plays a crucial role in other immune cells, even though the majority of research on the topic focuses on T cells and macrophages/monocytes. Human neutrophils, for instance, have intracellular stores of C3, CR1, and FB (2). C3 and FB are released from stores to trigger a local inflammatory process upon neutrophil activation. It is not yet known whether this process involves a traditional cascade reaction or simple proteolytic cleavage to release functional fragments, such as C3a or Ba (24, 39). M-ficolin/Ficolin-1, an activator of the LP pathway, was also found to be localized in neutrophils secretory granules (40).Neutrophil stimulation with C5a resulted in a quick release of the complement proteins FP, C3, and FB, which are critical for the assembly of the AP convertase C3bBb, linking a positive feedback loop between neutrophil and complement activation (41, 42). B cells also harbor a so-called B cell complosome (3), as mentioned above, B cells can transport C3(H2O) from the extracellular milieu into the cell and process it intracellularly *via* CTSL into C3b and C3a, however the biological implication of this observation remains to be established (20). The endogenous C3 expression is relatively low, and the serum is the main source of intracellular C3 in human B cells. According to research, both serum derived and pure C3 may enter the nucleus of live B cells, and its strong interaction with histone proteins and potential capacity to induce chromatin rearrangement implies that C3 might regulate DNA transcription *via* chromatin remodeling (43, 44). Natural killer cells (NK cells) express several complement receptors such as C3aR, CR3, CR4, C5aR1 and C5aR2, while C5aR1 and C5aR2 proteins were reported to be only expressed inside NK cells upon permeabilization. The intracellular expression of C5aR1 and C5aR2 was down-regulated in response to Poly (I: C) (a synthetic analogue of double-stranded RNA) or TNF-α (45). A similar pattern of intracellular C5aR1 and C5aR2 expression was also reported in CD3^+^CD56^+^ NKT-like cells (46). However, it is uncertain if this intracellular expression regulates NK cell activity or whether complement components such as C3 or C5 can be generated and secreted by the NK cell.

## 5 Complosome in non-immune cells

### 5.1 Cancer cells

Extensive studies have highlighted the importance of tumor cell-derived complosome (particularly C3/C3a) in cancer progression (47, 48). Intracellular activation of tumor cell-derived C3 inhibited CD8+ T cell infiltration and function by driving the accumulation and immune-suppressive activity of tumor-associated macrophages (TAMs) in a C3a-C3aR-dependent manner through the C3a-C3aR-PI3Kγ signaling pathway (49). More importantly, deletion of C3 in tumor cells that had high C3 expression enhanced the efficacy of anti–PD-L1 treatment (49). C3 downregulation by siRNA in ovarian cancer cells SKOV3ip1 inhibits tumor cell growth and migration (47). It has been shown that intracellular C3 in human colon carcinoma Caco2 cells can cleave cathepsin L and B and release C3a, just like CTSL can cleave C3 in CD4+ T cells in a non-C3 convertase manner, and C3a secretion can be downregulated by treatment with cathepsin L and B inhibitors in these cells (50). Other tumor cells, such as leukemia cells, express C3a and C5a receptors and respond to C3a and C5a stimulation by phosphorylation of p44/42 MAPK and AKT (51). Additionally, tumor cell derived-FB was found to be upregulated in pancreatic ductal adenocarcinoma (PDAC), promoting cancer cell proliferation by preventing cellular senescence and associated with myeloid-derived suppressor cells (MDSCs), immunosuppressive regulatory T-cells (Tregs), and TAMs in immunological tumor promotion (52). As previously stated, the complosme in CD4+ T cells and monocytes regulates basic metabolic processes and mTOR during T cell activation. Although evidence is lacking, it is very likely that the complosme functions similarly in tumor cells, which rely on aerobic glycolysis for cell survival, proliferation, and metastasis (48).

### 5.2 Other non-immune cells

There is also a growing number of reports about intracellular complement expression from stromal cells. For example, it have been demonstrated to regulate autophagy, energy management, and insulin production by pancreatic β cells (53, 54). Human pancreatic islets highly express intracellular C3, and C3 binds autophagy-related protein 16-1 (ATG16L1), thus regulating autophagy and contributing to β cell survival in human islet inflammation and diabetes (54). The C5b-C9 formation inhibitor protein, CD59, was demonstrated to be localized intracellularly in insulin granules in pancreatic β cells, thereby regulating insulin secretion by exocytosis (55, 56). Adipocytes can also produce complement components such as C3, C1q, properdin, and Components B and D, which have been linked to abnormal adipocyte function, immune cell infiltration, obesity, and insulin resistance (56, 57). Similarly to pancreatic β cells, intracellular C3 also protects human airway epithelial cells from stress-induced cell death (58). Intracellular C3 regulates Paneth cell turnover during repair of intestinal epithelial cells after injury (59). During mesenteric ischemia, C3 activation in a cathepsin-dependent manner in intestinal epithelial cells contribute greatly to intestinal tissue damage (50), confirming the pro-inflammatory role of intracellular C3. During SARS-CoV-2 infection, the complosome also plays an important role in lung epithelial cells by activating intracellular C3a, which can be blocked by a cell-permeable FB inhibitor, confirming the presence of an inducible cell-intrinsic C3 convertase in lung epithelial cells. Mechanically, complement gene transcription in lung epithelial cells was shown to be JAK1/2-STAT1–dependent (60). In retinal pigment epithelial (RPE) cells, intracellular expression of C3, C3a, C3aR, CR3, and FB was reported to be induced by internalized complement factor H-related 3 (FHR-3), in addition to proinflammatory cytokine secretion and inflammasome NLRP3 activation (61).

## 6 Conclusions and perspectives

Today, the complement system is defined as a complex network of fluid-phase, cell-surface-associated and intracellular proteins that act as pattern-recognition molecules, regulators, convertases, proteases, and signaling receptors that collectively mediate immunosurveillance and tissue homeostasis (62). The discovery of the complosome and its significant roles in metabolism, inflammatory response, and cancer progression (4, 63) in the last decade have brought fresh vitality into this field, but it has also generated more questions than answers. Several questions that need to be answered include: Where and how is the complosome activated and regulated/dysregulated? Is this cell specific? How does the complosome intersect with extracellular complement? What is the mechanism underlying the location–function of complement? How can the complosome be targeted therapeutically? Finding the answers to these questions will not only deepen our understanding of the complosome but also open up new insights into the development of complosome-targeted therapeutics.

## Author contributions

GY and FX conceived this study, SH and GY directed the study. FX, JG, and GY performed literature search. FX and JG drafted the manuscript. SH, ST, and GY provided critical intellectual revision. SH and GY provided financial support. All authors contributed to the article and approved the submitted version.
